# A Non-enzymatic Electrochemical Sensor for Glucose Detection Based on Ag@TiO_2_@ Metal-Organic Framework (ZIF-67) Nanocomposite

**DOI:** 10.3389/fchem.2020.573510

**Published:** 2020-10-15

**Authors:** Dooa Arif, Zakir Hussain, Manzar Sohail, Muhammad Arman Liaqat, Muzamil Ahmad Khan, Tayyaba Noor

**Affiliations:** ^1^School of Chemical and Materials Engineering (SCME), National University of Sciences & Technology (NUST), Islamabad, Pakistan; ^2^Department of Chemistry, School of Natural Sciences (SNS), National University of Sciences & Technology (NUST), Islamabad, Pakistan

**Keywords:** chronoamperometry, non-enzymatic glucose sensors, glucose oxidation, ZIF-67, electrochemical sensor

## Abstract

This work presents the preparation of an efficient and sensitive glucose sensor for the detection of glucose in an alkaline media. The glucose sensor is composed of a metal organic framework (MOF) composite comprising Ag@TiO_2_ nanoparticles. The hybrid of Ag@TiO_2_ encapsulated in ZIF-67 was synthesized by the solvothermal method and applied onto a glassy carbon electrode (GCE) for the non-enzymatic sensing of glucose. The porosity of ZIF-67 was favorable for the unhindered diffusion and entrapment of glucose and its cavities served as reaction vessels. The electrochemical behavior of Ag@TiO_2_@ZIF-67 showed amplified results when compared with that of Ag@TiO_2_ and ZIF-67. Cyclic tests toward the oxidation of glucose has demonstrated excellent stability of a MOF-based hybrid sensor. The sensor based on Ag@TiO_2_@ZIF-67 showed high sensitivity of 0.788 μAμM^−1^cm^−2^ with a linear concentration range of 48 μM^−1^ mM and a response time of 5 s with an excellent detection limit of 0.99 μM (S/N = 3).

## Introduction

Diabetes mellitus is a very common and fatal disease. Millions of people are affected around the world by this chronic metabolic disorder. A recent study by the World Health Organization (WHO) and the International Diabetes Federation (IDF) revealed that ~422 million people are diabetic and by 2030 this number will reach to 642 million. Besides remedies to control blood glucose levels, development of accurate, reliable, and precise sensors to measure blood glucose is ever increasing and essential in controlling diabetes. While looking at such high numbers for blood glucose detection, ~85% of the biosensors are analytical devices (Dhara and Mahapatra, 2018).

Commercially available disposable glucose biosensors depend on the catalytic activity of an immobilized enzyme where a systematic and quick cyclic regeneration process of an enzyme is the foremost criteria for catalytic activity (Taylor et al., 1995). Due to instability of the enzyme-based biosensors, development of non-enzymatic sensors is crucial where non-biological catalysts such as functionalized nanomaterials as immobilization platforms can be exploited (Bai et al., 2008; Rathod et al., 2010).

The promising fourth generation glucose sensors based on metal oxides and nanostructured metals, used as electrodes to perform the electro-oxidation of glucose directly, producing substantial electrocatalytic activity toward glucose (Bai et al., 2010; Xu et al., 2010). Large number of reactions can be catalyzed using noble metal nanoparticles (NPs) with a lower activation barrier due to extraordinary catalytic properties. Metals such as Pt, Au, Pd, and their NPs as well as metal oxides and sulfides of Zn, Ni, Mn, Co, Fe, and Cu have been extensively studied for glucose oxidation in particular and for their enhanced catalytic properties in general (Liu et al., 2009; Mei et al., 2016). Noble metal-modified electrodes show increased electron transfer rates due to higher conductivity, electrocatalytic properties, and large surface areas. Also, size and morphology of the NPs of such noble metals greatly influence their electrocatalytic properties and such NPs can be exploited to lower over potentials for redox reactions (Pal and Ganesan, 2009; Azad and Ganesan, 2010; Rastogi et al., 2014; Gupta and Ganesan, 2015; Sonkar and Ganesan, 2015). Furthermore, to prepare an efficient electrocatalytic sensor, NPs are usually immobilized on solid supports such as metal organic frameworks (MOFs) (Zhu et al., 2017). Incorporating metal nanoparticles into the MOF structure not only increases the catalytic activity but also increases the functional sites (Yadav et al., 2016). Ag NPs have many unique characteristics when compared to other noble metal nanoparticles such as Ru and Pt. For instance, Ag possesses high conductivity and biocompatibility which increases its demand in the field of sensing. Ag NPs also increase specific surface areas and enhance mass transport ability in electrochemical sensors.

Among various metal oxides for electrocatalytic sensors, TiO_2_ nanostructures have gained greater attention due to their high surface area, non-toxicity, electrocatalysis, oxygen storage capacity, and biocompatibility but their use remains limited due to low conductivity (Lu et al., 2012). To circumvent this limitation, nanocomposites of TiO_2_ with various metal NPs have been explored for non-enzymatic glucose (NEG) sensors. To this end, Ag due to its high electron conductivity, can be considered as the metal of choice together with TiO_2_ to amplify the electro catalytic activity for glucose oxidation (Ansari et al., 2015). Nanocomposite structures incorporating Ag doped TiO_2_ have gained recent attention, also due to their potential for chemical and biological sensing (Liang et al., 2011). Ag@TiO_2_ nanoparticles played an important role in facilitating the electron transfer by providing specific large surface areas to glucose and also help in the absorption of glucose (Dayakar et al., 2018). In order to study the metal NPs doped metal oxides for electrocatalytic activity, material has to be deposited on a support where the use of MOFs has gained huge attention recently (Ahmed and Jhung, 2014).

Recently, various MOF-based nanocomposites have been reportedly used as sensing elements with excellent stability in harsh environments (highly acidic/basic conditions) as well as having an enhanced catalytic performance and the provision of a facilitated path for signal transduction (Liu et al., 2016; Yin et al., 2017). Different nanocomposites of MOFs with metal nanoparticles, quantum dots, polymers, polyoxometalates, carbon nanotubes, enzymes, and biomolecules have been reported in the recent literature (Zhu and Xu, 2014). Owing to its large surface areas, porous structure, and easily modifiable surface properties MOFs when used for electrochemical sensing should possess important characteristics such as electrical conductivity, electrochemical activity, water stability, and biocompatibility. Although pristine MOFs exhibit remarkable catalytic properties, the incorporation of metal nanoparticles into the MOF structures demonstrate synergistic effects. High porosity of MOFs enable them to anchor onto or encapsulate functional metal nanoparticles (MNPs) within their cavities forming MNPs@MOF composites. The addition of noble metal nanoparticles enhance the catalytic activity of MOF by exhibiting a synergistic effect and in turn MOFs prevent aggregation of nanoparticles and provide more catalytic sites for glucose oxidation (Liu et al., 2020).

Due to porous structures, MOFs and their composites demonstrate superior electrochemical redox properties and excellent electrocatalytic properties. Besides porosity, such structures also demonstrate facilitated transport of ions across structures with huge permeability to promote fast internal diffusion via phase transformation (Doménech et al., 2007). Therefore, multi-functional MOF-based composites show huge potential to be further explored for electrochemical sensing. To this end, a number of MOF-based composites have been produced and explored for electrochemical applications, including GO-MOFs, CNTs-MOFs, metal oxide NPs-MOFs, Au NPs-MOFs, and Ag NPs-MOFs (Song et al., 2016). Some of the developed MOF-based nanocomposites have also been explored for glucose sensing. In a recent study, Co-MOF nanosheets on nickel foam were used to serve as a superior sensor for non-enzymatic glucose detection (Li et al., 2019). Similarly, encapsulated CuNPs in ZIF-8 have been investigated to show outstanding performance in electrocatalysis and glucose sensing (Shi et al., 2016).

For non-enzymatic glucose sensing, Ag@ZIF-67 has also been studied and modified electrodes have demonstrated high performance toward glucose detection (Meng et al., 2018). ZIF-67 shows superior properties among other MOFs such as exceptional thermal and chemical stabilities and good selectivity for target analytes (Wang et al., 2018). ZIFs belong to a well-known family of MOFs and due to their unique characteristics such as large surface area and high porosity; they are used in various applications such as catalysis and sensing. However, they lack good electrical conductivity. In order to use ZIF-67 in electrochemical applications, several strategies are introduced by researchers such as encapsulating active guests and adding conductive coating (Zhu et al., 2020).

With the above in view, non-enzymatic detection of glucose by exploiting noble metal doped metal oxides with a porous support such as MOFs is the most attractive research area to be further explored for a reliable, sensitive, and selective sensing of glucose (Lü et al., 2014; Zhou et al., 2017). Hence, in the present contribution, we have synthesized and investigated Ag@TiO_2_ NPs encapsulated in ZIF-67 for the electrochemical sensing of glucose. The obtained Ag@TiO_2_@ZIF-67 sensor showed enhanced electrocatalytic activity toward glucose without any enzyme.

## Experimental

### Chemicals

2-Methylimidazole, D(+)-Glucose anhydrous, and L (+)-Ascorbic acid were purchased from Merck (Germany) while Co(NO_3_)_2_·6H_2_O was purchased from ROTH (Germany). Silver nitrate was purchased from the Duksan Pure Chemicals Co. Ltd. (S. Korea). Uric acid and methanol were procured from Sigma Aldrich (Germany). All chemicals were of analytical grade and used without further purification. All experiments were carried out using ultrapure water.

### Synthesis of ZIF-67

The already reported procedure was followed to synthesize ZIF-67 at room temperature (Zhou et al., 2017). Briefly, metal salt [Co (NO_3_)_2_.6H_2_O, 1 mmol, 0.291 g] and ligand (2-methylimidazolate, 8 mmol, 0.66 g) were mixed in methanol separately followed by the addition of the metal solution into the ligand solution drop wise where a change in color was observed instantly. After complete addition, the solution was vigorously stirred for the next 24 h. The precipitate formed was separated using a centrifuge (4,000 rpm for 10 min) and washed three times with methanol. The obtained product was placed in a vacuum oven for overnight drying at 60°C (Zhou et al., 2017).

### Synthesis of Ag@TiO_2_

Synthesis of TiO_2_ NPs was carried out according to the already reported method (Saleem and Habib, 2016). Briefly, titanium (IV) isopropoxide (TTIP), ethylene glycol monomethyl ether, and ethanolamine with a molar ratio of 1:4:0.5 were mixed in a round bottom flask and stirred for 1 h at room temperature followed by stirring at 80°C for 1 h and at 120°C for an additional 2 h. The whole process was carried out in an inert environment. The appearance of a yellowish-orange color confirmed the formation of sol which was calcined at 500°C for 6 h to obtain TiO_2_ NPs.

For the synthesis of Ag doped TiO_2_ NPs, first AgNO_3_ was added to ethylene glycol monomethyl ether in a round bottom flask, stirred for 10 min followed by the addition of TTIP and ethanolamine to this solution with the same molar ratio as mentioned above. The mixture was again stirred under various heating conditions as mentioned above. The appearance of a dark color confirmed the formation of sol which was calcined at 500°C for 6 h to obtain the Ag doped TiO_2_ NPs.

### Synthesis of Ag@ZIF-67

For the synthesis of Ag@ZIF-67, ZIF-67 was pre-heated for 5 h under vacuum at 100°C. Following the procedure, 700 mg ZIF-67 was dispersed in ethanol (11 mL) followed by the addition of AgNO_3_ (0.006 mmol in ethanol) into the ZIF-67 solution drop wise with constant stirring and stirring continued for another 5 h. The product obtained was washed thoroughly with ethanol by centrifugation and dried under vacuum at 80°C for 12 h. The obtained sample was dispersed again in ethanol followed by the addition of NaBH_4_ (0.06 mmol) to obtain a purple black product after washing and centrifugation (Meng et al., 2018).

### Synthesis of TiO_2_@ZIF-67 and Ag@TiO_2_@ZIF-67

For the synthesis of Ag@TiO_2_@ZIF-67, Ag@TiO_2_ (14.9 mg) was added to the precursor solution of (Co (NO_3_)_2_.6H_2_O in methanol, 0.873 g) with the 2-methylimidazolate (1.98 g) in methanol (ligand solution) and left for stirring for 10 min. Following the reaction, the solution was transferred to stainless steel Teflon lined bomb vessel and placed in an oven for 5 h at 150°C to obtain a precipitate which was washed with ethanol three times and vacuum dried for 12 h to obtain the final product. TiO_2_@ZIF-67 was also synthesized by following the procedure mentioned for the synthesis of Ag@TiO_2_@ZIF-67.

### Fabrication of the Modified Electrode

Before modifying the glassy carbon electrode (GCE) with the samples, its pre-polishing was carried out with an alumina slurry followed by washing with ethanol and distilled water alternatively and finally sonication for 5 min. Ag@TiO_2_@ZIF-67 (2 mg) was dispersed into distilled water (1.0 mL), ethanol (40 μL), and 0.5 wt % nafion (10 μL) and sonicated for 1 h to prepare a homogeneous suspension. Then the suspension (10 μL) was sequentially drop casted onto GCE. After coating, GCE was set for drying overnight. The similar procedure was followed for the electrochemical testing of ZIF-67, Ag@ZIF-67, and TiO_2_@ZIF-67. The modified electrodes were dried overnight for carrying out electrochemical tests. No particular activation was required to start sensing glucose with the electrode.

### Characterization

The surface morphology of the prepared materials was observed through SEM (JEOL JSM-6042 A; Japan) while structural studies were carried out by x-ray diffraction (XRD, STOE- Seifert X'Pert PRO), using CuKα radiations (λ = 1.5406°A) at an angle (2θ) ranging from 20° to 80°. Electrochemical behavior of the fabricated electrodes was measured using the Gamry G750 electrochemical workstation with the standard three-electrodes system comprised of GCE, Pt-wire, and Ag/AgCl acting as the working electrode, counter electrode, and reference electrode, respectively. For the determination of the electrochemical behavior of the modified electrodes, cyclic tests and chronoamperometry were performed. The electrochemical behavior of the modified electrodes was evaluated by cyclic voltammetry and chronoamperometry.

## Results and Discussion

### XRD Analysis

**Figure 1:**
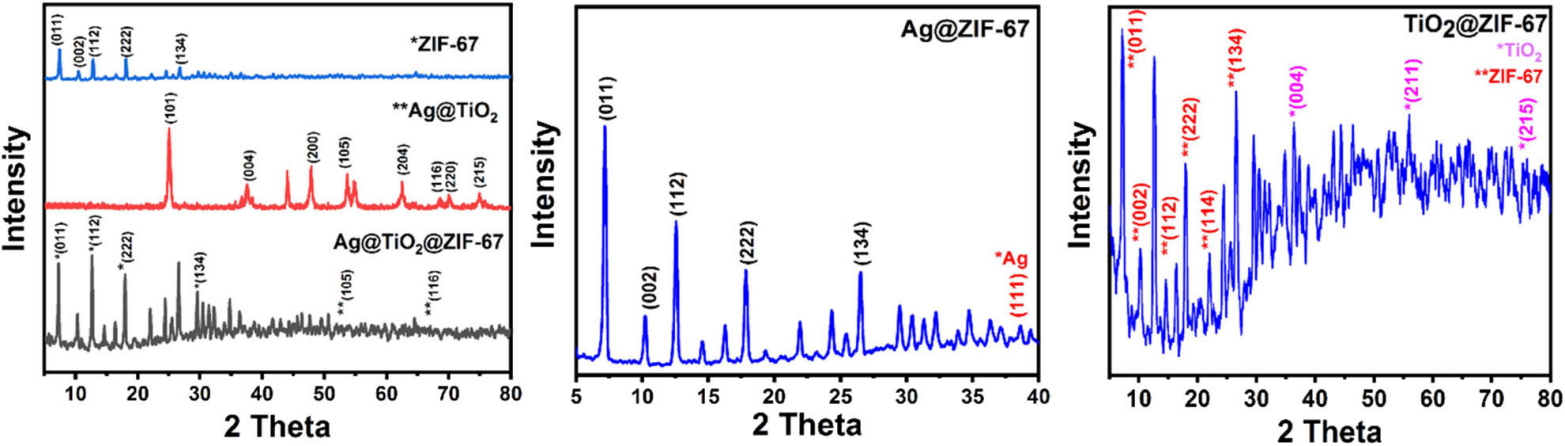
XRD patterns of **(A)** ZIF-67, Ag@TiO_2_, and Ag@TiO_2_@ZIF-67; **(B)** Ag@ZIF-67; and **(C)** TiO_2_@ZIF-67.

X-ray diffraction patterns of the prepared samples are shown in Figure 1. ZIF-67 (Figure 1A) shows sharp reflections at 2 theta = 7.4°, 10.4°, 12.7°, 14.8°, 18°, 22.1°, 26.7° corresponding to (011), (002), (112), (022), (222), (134) crystal planes, respectively (Banerjee et al., 2008). In the case of XRD patterns of Ag@TiO_2_ (Figure 1A) peaks can be observed at 25.23°, 37.8°, 48°, 53.9°,62.5°,68.8°, 70°,75° corresponding to (101), (004), (200), (105), (204), (116), (220), (215) crystal planes of TiO_2_ demonstrating anatase crystallization (Dayakar et al., 2018). In the case of Ag@TiO_2_@ZIF-67 (Figure 1A), all the diffraction peaks for ZIF-67 could be seen after the successful addition of Ag@TiO_2_, demonstrating retention of ZIF-67 crystal structure in the nanocomposite. Furthermore, characteristic peaks of Ag@TiO_2_ [e.g., 53.9° (105) and 68.8° (116)] could be easily observed due to the presence of no peaks for ZIF-67 in the scan rate of 40° to 80°. Similarly, from the XRD patterns of Ag@ZIF-67 (Figure 1B) no sharp peaks for Ag could be seen due to the minute quantity of Ag NPs in the MOFs. However, a single peak at 38.2° indexed to the (111) crystal face of Ag could be seen. Therefore, it can be assumed that the addition of Ag NPs in the MOF has no effect on its crystal structure. Finally, in the XRD patterns of TiO_2_@ZIF-67 (Figure 1C), peaks of TiO_2_ were observed within the scan rate of 30°-80° contrary to similar reflections due to Ag NPs in the previous case, also due to larger quantity of TiO_2_ in comparison to Ag NPs in the MOF.

### SEM Analysis

**Figure 2:**
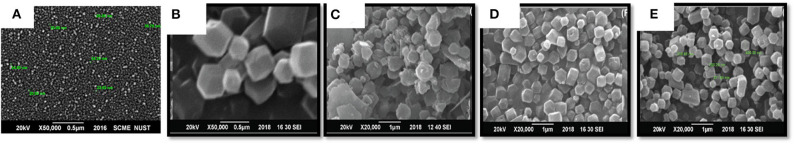
SEM of **(A)** Ag@TiO_2_; **(B)** ZIF-67; **(C)** Ag@ZIF-67; **(D)** TiO_2_@ZIF-67; and **(E)** Ag@TiO_2_@ZIF-67.

Scanning electron microscope (SEM) analysis of all the prepared samples is given in Figure 2. In the case of ZIF-67, the structure is a well-defined dodecahedron with an average size of 350 nm. Similarly, Ag@TiO_2_@ZIF-67 maintains its morphology, as shown in the Figure 2E. The addition of nanoparticles in ZIF-67 does not affect the structure of the MOF nanoparticles composite. Ag@TiO_2_ nanoparticles could not be seen in the SEM micrograph of Ag@TiO_2_@ZIF-67; considering that, they most likely reside in the pores of ZIF-67.

### Electrochemical Behavior of Modified Electrodes

**Figure 3:**
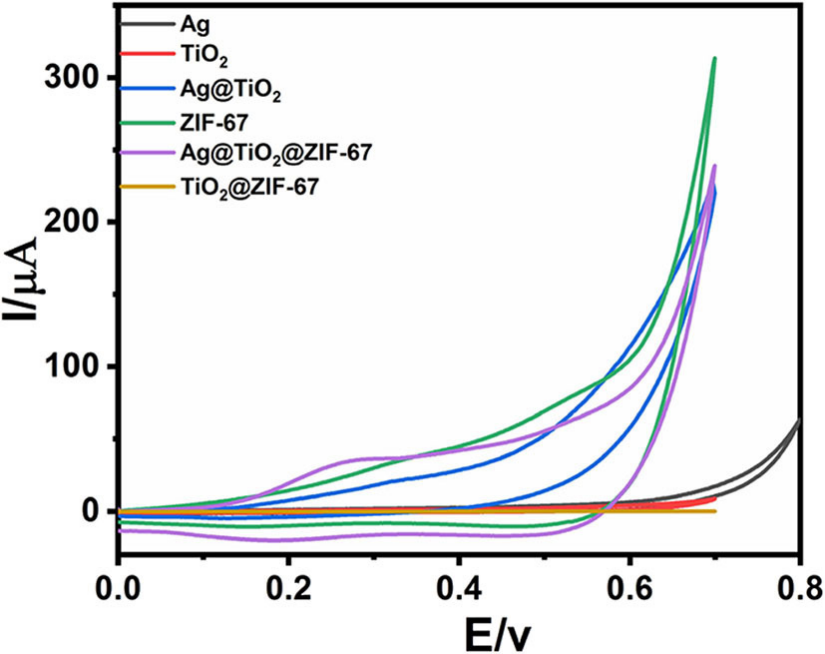
CVs of GCE, Ag NPs, ZIF-67, Ag@TiO_2_, Ag@ZIF-67, TiO_2_@ZIF-67, and Ag@TiO2@ZIF-67 in the absence of glucose at a scan rate of 50 mVs^−1^.

**Figure 4:**
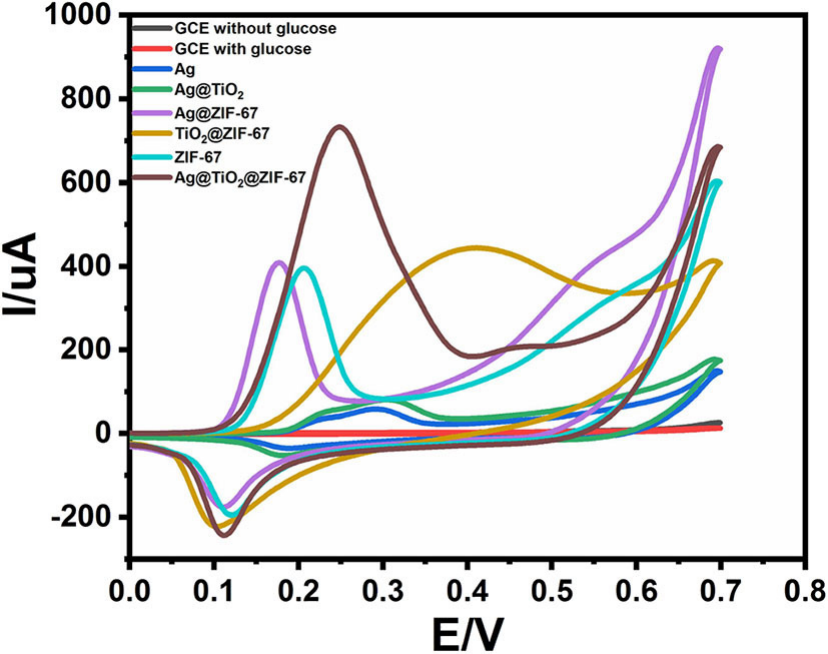
CVs of GCE, Ag NPs, ZIF-67, Ag@TiO_2_, Ag@ZIF-67, TiO_2_@ZIF-67, and Ag@TiO2@ZIF-67 in the presence of 2 mM glucose at a scan rate of 50 mVs^−1^.

The cyclic voltammetry (CV) of bare GCE, Ag NPs, ZIF-67, Ag@TiO_2_, Ag@ZIF-67, TiO_2_@ZIF-67, and Ag@TiO_2_@ZIF-67 were carried out in a 0.1 M NaOH electrolytic solution having glucose (2 mM) at scan rate 50 mVs^−1^. Figure 3 shows CVs of GCE, Ag NPs, ZIF-67, Ag@TiO_2_, Ag@ZIF-67, TiO_2_@ZIF-67, and Ag@TiO_2_@ZIF-67 in the absence of glucose at a scan rate of 50 mVs^−1^. Due to having no catalytic activity toward glucose, bare GCE showed no redox peak in the presence and absence of glucose (Figure 4). Similarly, Ag NPs showed a peak current of 58.00 μA at 0.365 V, Ag@TiO_2_ at 80.40 μA at 0.337 V, Ag@ZIF-67 at 408.9 μA at 0.215 V, and TiO_2_@ZIF-67 at 444.4 μA at 0.243 V. Finally, CV of the Ag@TiO_2_@ZIF-67 decorated GCE demonstrated increased current response of 732.8 μA at 0.189 V as compared to pristine MOF which showed a current response of 395.9 μA at 0.207 V. Obtained results clearly demonstrated that catalytic activity increased with the addition of Ag@TiO_2_ NPs in the ZIF-67. MOF provides a specific large surface area, important for efficient electron transfer, which can be observed from the above given current response values. It is clearly visible from the cyclic voltammetry graphs that Ag@TiO_2_@ZIF-67 modified GCE showed a much higher current response as compared to Ag and Ag@TiO_2_ separately. Ag@TiO_2_@ZIF-67 nanocomposites exhibit a better electrochemical response indicating that conductive nanomaterials such as Ag@TiO_2_ when incorporated into MOF enhance the electron transfer reaction. Ag@TiO_2_ provides active sites and MOF acts as solid support for the effective loading of Ag@TiO_2_.

Catalytic activity depends on a number of factors such as particle size, specific surface area, and presence of metals on the surface providing active sites for the electrical signal transmission and redox reactions (Dayakar et al., 2018). In the present case, electrode performance was overall improved toward glucose detection by combining MOFs with Ag@TiO_2_ NPs. Glucose electro-oxidation occurs due to the existence of Co (II) that intensifies the anodic current. The equation below shows the general mechanism of glucose oxidation.

$$\begin{array}{r} \text{Co}\left(\text{II}\right)-\text{MOF}+\text{O}{\text{H}}^{-}\text{Co}\left(\text{III}\right)\to \text{MOF}+{\text{e}}^{-}\\ \text{Co}\left(\text{III}\right)-\text{MOF}+\text{O}{\text{H}}^{-}+\text{GlucoseMOF}\to \text{Gluconolactone}\\ +{\text{H}}_{2}\text{O}+{\text{e}}^{-} \end{array}$$

**Figure 5:**
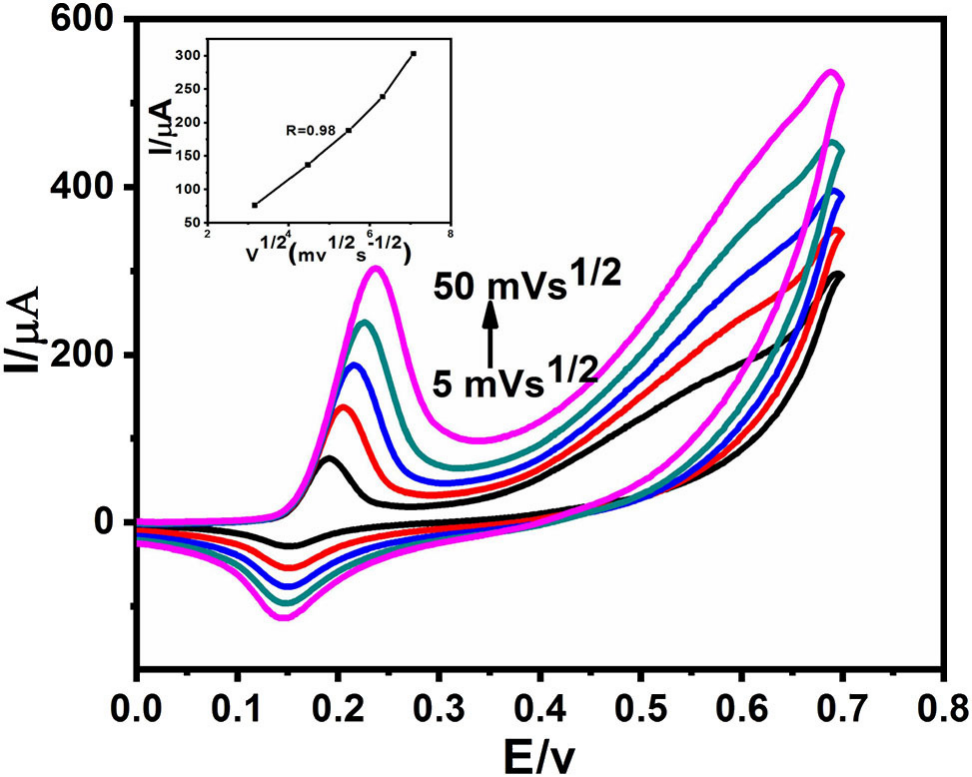
CVs of Ag@TiO_2_@ZIF-67 in 0.1 M NaOH with 2 mM glucose at different scan rates from 5 to 50 mVs^−1^. Inset: of the current vs. the square root of the scan rate.

The measurement of CVs at different scan rates is shown in Figure 5. It can be seen that with an increase in the potential scan rate, the current of anodic and cathodic peak increased distinctly. For the ideal detection of glucose, the electrochemical kinetics should be a typically diffusion-controlled electrochemical process, depicted in the Figure 5 inset graph, the linear correlation between the square root of the scan rate and both the anodic and cathodic current peaks (Yang et al., 2015). There is a slight deviation in the linearity at a scan rate of 50 mVs^−1^. This suggests that either it is electrochemically quasi reversible or maybe electron transfer occurs through surface-adsorbed species (Elgrishi et al., 2018).

### Amperometric Detection of Glucose on Ag@TiO_2_@ZIF-67

Sensitivity along with less signal to noise ratio is an important factor for sensing applications that can be assessed by the amperometric method, a well-known method that has convective mass transport and can detect glucose rapidly as compared to other glucose detection methods (Dayakar et al., 2018). In the present case, 0.4 V applied potential was optimized for chronoamperometry. Applied potential is the voltage value on which sensing material shows its oxidation peak. The applied potential is optimized by checking the range of different potential values such as +0.38, +0.35, and +0.4 V. By increasing working potential, a high current response was obtained. Therefore, 0.4 V was selected as the best applied potential.

**Figure 6:**
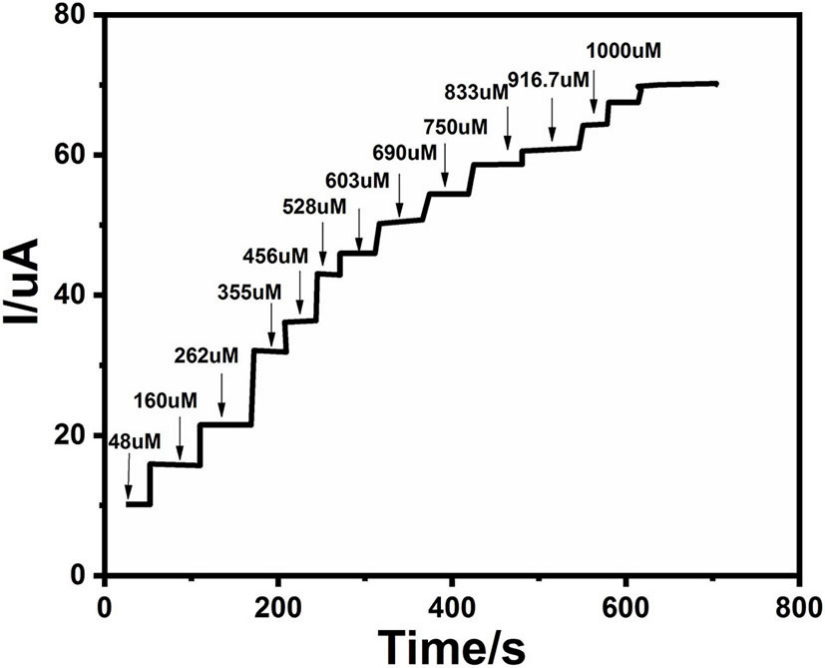
Chronoamperometric response of various glucose concentrations at the Ag@TiO_2_@ZIF-67 modified GCE in 0.1 M NaOH solution. The current increases upon increasing glucose concentrations.

**Figure 7:**
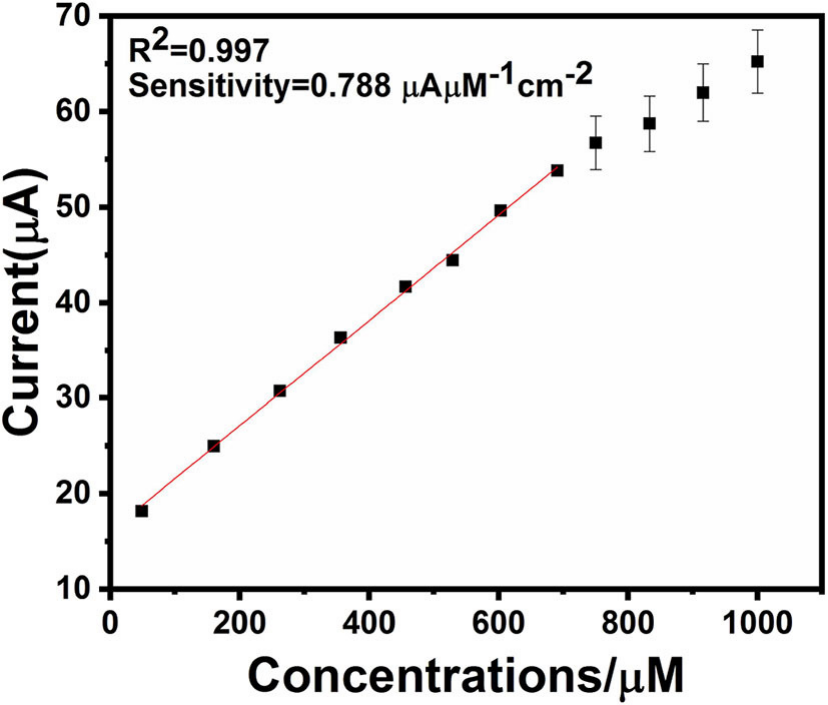
Scatter plot of current density vs. added glucose concentration.

An electrochemical sensor's performance depends on applied potential and it is significant to obtain the ideal performance for glucose detection (Zhang et al., 2016). Figure 6 shows an ephemeral response of Ag@TiO_2_@ZIF-67 in the presence of 0.1 M NaOH on consecutive addition of different glucose concentrations with constant stirring and a working potential of 0.4 V. With successive glucose addition, a stepwise increased current response was obtained within 5 s, which signifies the facile electron current response due to the Ag@TiO_2_@ZIF-67/GCE electrode. Figure 7 represents a good linear relationship of electrocatalytic activity and various glucose concentrations in the range of 48 μM to 1 mM having a correlation coefficient of 0.997. MOF nanocomposites provide enough catalytic sites for the oxidation of glucose on reaching 1 mM glucose concentration. All the catalytic sites were saturated at this concentration, leaving not enough sites free for the higher concentration of glucose. As a result sensitivity gradually decreased with the increase in the glucose concentration. Sensitivity can be calculated by a calibration curve obtained from the amperometric response curve. An Ag@TiO_2_@ZIF-67 based electrochemical sensor showed a sensitivity of 0.788 μAμM^−1^cm^−2^ and a detection limit of 0.99 μM. These values are also compared with the MOF nanocomposites in Table 1.

**Table 1:** Comparison of sensitivity of different glucose sensors.

**Modified electrode**	**Sensitivity**	**LOD (μM)**	**References**
Cu-in-ZIF-8	0.412 μAμM^−1^cm^−2^	2.76 μM	Shi et al., 2016
Co-MOF/NF	10.886 μA μM^−1^cm^−2^	1.3 nM	Li et al., 2019
Cu_3_(BTC)_2_-derived CuO nanorod	1.523.5 μA μM^−1^cm^−2^	1 μM	Kim et al., 2019
HKUST_3_-1/KSC800	28.67 μA μM^−1^cm^−2^	0.48 μM	Xie et al., 2018
ZIF-N2	0.227 μA μM^−1^cm^−2^	5.69 μM	Shi et al., 2017
Ni-MOF/CNTs	13.85 μA μM^−1^cm^−2^	0.82 μM	Zhang et al., 2018
GS@ZIF-67	1.521.1 μA μM^−1^cm^−2^	0.36 μM	Chen et al., 2019
Amorphous derivative of ZIF-67	1.074.22 μA μM^−1^cm^−2^	3.9 × 10^−6^M	Zhou et al., 2020
Cu_2_O@ZIF-67	181.34 μA μM^−1^cm^−2^	6.5 μM	Yang et al., 2020
ZIF-67	0.152 μAμM^−1^cm^−2^	1.6 μM	Meng et al., 2018
Ag-0.5%@ZIF-67/GCE	0.379 μAμM^−1^cm^−2^	0.6 μM	Meng et al., 2018
Ag@TiO_2_@ZIF-67	0.788 μAμM^−1^cm^−2^	0.99 μM	This work

### Selectivity of the Sensor

**Figure 8:**
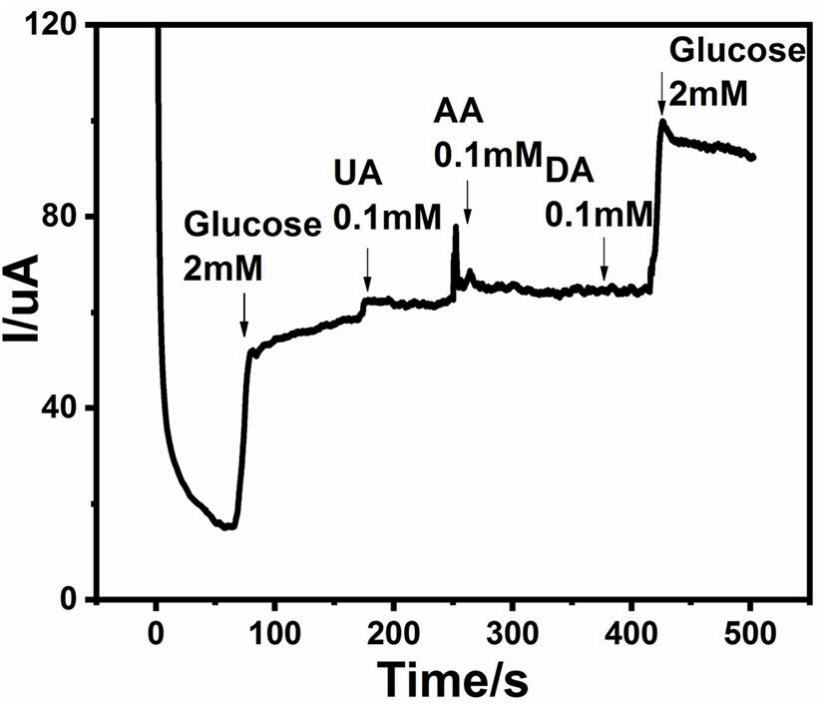
Chronoamperometric response of Ag@TiO_2_@ZIF-67/GCE on sequential addition of 2 mM glucose, 0.1 mM uric acid (UA), 0.1 mM ascorbic acid (AA), and 0.1 mM dopamine (DA). Applied potential: +0.4 V.

Blood glucose level normally lies within the range of 4–7 mM and varies from person to person because of physiological and structural differences among them. Along with glucose, there exists other interfering species, but 30–50 times less as compared to glucose (Cai et al., 2014). It is important to assess the selectivity of a non-enzymatic glucose sensor, because the presence of interfering agents affect the detection of glucose. Generally, interfering species like dopamine, ascorbic acid (AA), uric acid (UA), and acetaminophen (AP) coexist with glucose in biological systems. A response curve of selectivity is shown in Figure 8. It can be observed that when 2 mM of glucose was introduced into the alkaline solution, an obvious current peak appeared. Then 0.1 mM UA, 0.1 mM AA, and 0.1 mM DA were added sequentially, which showed minimal response as compared to glucose. It is clear from the given response that an Ag@TiO_2_@ZIF-67 modified electrode has good selectivity toward glucose.

## Conclusion

In the present contribution, we have demonstrated a non-enzymatic electrochemical sensor based on an Ag@TiO_2_@ZIF-67 MOF composite for the detection of glucose. The present study mainly focused on demonstrating the synergistic effect of Ag@TiO_2_ and ZIF-67 in developing a glucose sensor. By adding Ag@TiO_2_ into the MOF structure, the surface structure and subsequent electrochemical property of the working electrode changed, resulting in an enhanced performance. Our developed material and fabricated electrode is highly sensitive and selective within a limit of detection of 0.99 (μM) for glucose.

## Data Availability Statement

The original contributions presented in the study are included in the article/supplementary material, further inquiries can be directed to the corresponding author.

## Author Contributions

DA: data collection and manuscript write up. ZH: conceptualization, supervision, and manuscript editing. MS: electrochemistry and manuscript correction. ML and MK: synthesis of nanoparticles and characterization of materials. TN: MOF synthesis and characterization. All authors contributed to the article and approved the submitted version.

## Conflict of Interest

The authors declare that the research was conducted in the absence of any commercial or financial relationships that could be construed as a potential conflict of interest.

## References

[B1] Ahmed I. Jhung S. H. (2014). Composites of metal–organic frameworks: preparation and application in adsorption. Mater. Today 17, 136–146. 10.1016/j.mattod.2014.03.002

[B2] Ansari S. A. Khan M. M. Ansari M. O. Cho M. H. (2015). Gold nanoparticles-sensitized wide and narrow band gap TiO_2_ for visible light applications: a comparative study. New J. Chem. 39, 4708–4715. 10.1039/C5NJ00556F

[B3] Azad U. P. Ganesan V. (2010). Influence of metal nanoparticles on the electrocatalytic oxidation of glucose by Poly(Ni^II^teta) modified electrodes. Electroanalysis 22, 575–583. 10.1002/elan.200900435

[B4] Bai H. Han M. Du Y. Bao J. Dai Z. (2010). Facile synthesis of porous tubular palladium nanostructures and their application in a nonenzymatic glucose sensor. Chem. Commun. 46:1739. 10.1039/b921004k 20177634

[B5] Bai Y. Sun Y. Sun C. (2008). Pt–Pb nanowire array electrode for enzyme-free glucose detection. Biosens. Bioelectron. 24, 579–585. 10.1016/j.bios.2008.06.003 18619831

[B6] Banerjee R. Phan A. Wang B. Knobler C. Furukawa H. O'Keeffe M.. (2008). High-throughput synthesis of zeolitic imidazolate frameworks and application to CO_2_ capture. Science 319, 939–943. 10.1126/science.1152516 18276887

[B7] Cai B. Zhou Y. Zhao M. Cai H. Ye Z. Wang L. (2014). Synthesis of ZnO–CuO porous core–shell spheres and their application for non-enzymatic glucose sensor. Appl. Phys. A Mater. Sci. Process. 118, 989–996. 10.1007/s00339-014-8855-8

[B8] Chen X. Liu D. Cao G. Tang Y. Wu C. (2019). *In situ* synthesis of a sandwich-like graphene@ZIF-67 heterostructure for highly sensitive nonenzymatic glucose sensing in human serums. ACS Appl. Mater. Interfaces 11, 9374–9384. 10.1021/acsami.8b22478 30727733

[B9] Dayakar T. Rao K. V. Bikshalu K. Rajendar V. Park S. H. (2018). Novel synthesis and characterization of Ag@TiO_2_ core shell nanostructure for non-enzymatic glucose sensor. Appl. Surf. Sci. 435, 216–224. 10.1016/j.apsusc.2017.11.077

[B10] Dhara K. Mahapatra D. R. (2018). Electrochemical nonenzymatic sensing of glucose using advanced nanomaterials. Microchim. Acta 185:49. 10.1007/s00604-017-2609-1 29594566

[B11] Doménech A. García H. Doménech-Carbó M. T. Llabrés-i-Xamena F. (2007). Electrochemistry of metal–organic frameworks: a description from the voltammetry of microparticles approach. J. Phys. Chem. C 137, 13701–13711. 10.1021/jp073458x

[B12] Elgrishi N. Rountree K. J. McCarthy B. D. Rountree E. S. Eisenhart T. T. Dempsey J. L. (2018). A practical beginner's guide to cyclic voltammetry. J. Chem. Educ. 95, 197–206. 10.1021/acs.jchemed.7b00361

[B13] Gupta R. Ganesan V. (2015). Gold nanoparticles impregnated mesoporous silica spheres for simultaneous and selective determination of uric acid and ascorbic acid. Sens. Actuat B Chem. 219, 139–145. 10.1016/j.snb.2015.05.018

[B14] Kim K. Kim S. Lee H. N. Park Y. M. Bae Y. S. Kim H. J. (2019). Electrochemically derived CuO nanorod from copper-based metal-organic framework for non-enzymatic detection of glucose. Appl. Surf. Sci. 479, 720–726. 10.1016/j.apsusc.2019.02.130

[B15] Li Y. Xie M. Zhang X. Liu Q. Lin D. Xu C. (2019). Co-MOF nanosheet array: A high-performance electrochemical sensor for non-enzymatic glucose detection. Sensors Actuators B Chem. 278, 126–132. 10.1016/j.snb.2018.09.076

[B16] Liang Y. Q. Cui Z. D. Zhu S. L. Liu Y. Yang X. J. (2011). Silver nanoparticles supported on TiO2 nanotubes as active catalysts for ethanol oxidation. J. Catal. 278, 276–287. 10.1016/j.jcat.2010.12.011

[B17] Liu C. Sen L.i J. Pang H. (2020). Metal-organic framework-based materials as an emerging platform for advanced electrochemical sensing. Coord. Chem. Rev. 410:213222 10.1016/j.ccr.2020.213222

[B18] Liu X. Hu Q. Wu Q. Zhang W. Fang Z. Xie Q. (2009). Aligned ZnO nanorods: A useful film to fabricate amperometric glucose biosensor. Colloids Surfaces B Biointerfaces 74, 154–158. 10.1016/j.colsurfb.2009.07.011 19660919

[B19] Liu X.-W. Sun T.-J. Hu J.-L. Wang S.-D. (2016). Composites of metal–organic frameworks and carbon-based materials: preparations, functionalities and applications. J. Mater. Chem. A 4, 3584–3616. 10.1039/C5TA09924B

[B20] Lu X. Wang G. Zhai T. Yu M. Gan J. Tong Y.. (2012). Hydrogenated TiO _2_ Nanotube Arrays for Supercapacitors. Nano Lett. 12, 1690–1696. 10.1021/nl300173j 22364294

[B21] Lü Y. Zhan W. He Y. Wang Y. Kong X. Kuang Q.. (2014). MOF-templated synthesis of porous Co_3_O_4_ concave nanocubes with high specific surface area and their gas sensing properties. ACS Appl. Mater. Interfaces 6, 4186–4195. 10.1021/am405858v 24559195

[B22] Mei H. Wu W. Yu B. Wu H. Wang S. Xia Q. (2016). Nonenzymatic electrochemical sensor based on Fe@Pt core–shell nanoparticles for hydrogen peroxide, glucose and formaldehyde. Sensors Actuators B Chem. 223, 68–75. 10.1016/j.snb.2015.09.044

[B23] Meng W. Wen Y. Dai L. He Z. Wang L. (2018). A novel electrochemical sensor for glucose detection based on Ag@ZIF-67 nanocomposite. Sensors Actuators B Chem. 260, 852–860. 10.1016/j.snb.2018.01.109

[B24] Pal M. Ganesan V. (2009). Zinc phthalocyanine and silver/gold nanoparticles incorporated mcm-41 type materials as electrode modifiers. Langmuir 25, 13264–13272. 10.1021/la901792b 19824690

[B25] Rastogi P. K. Ganesan V. Krishnamoorthi S. (2014). A promising electrochemical sensing platform based on a silver nanoparticles decorated copolymer for sensitive nitrite determination. J. Mater. Chem. A 2, 933–943. 10.1039/C3TA13794E

[B26] Rathod D. Dickinson C. Egan D. Dempsey E. (2010). Platinum nanoparticle decoration of carbon materials with applications in non-enzymatic glucose sensing. Sensors Actuators B Chem. 143, 547–554. 10.1016/j.snb.2009.09.064

[B27] Saleem H. Habib A. (2016). Study of band gap reduction of TiO2 thin films with variation in GO contents and use of TiO_2_/Graphene composite in hybrid solar cell. J. Alloys Compd. 679, 177–183. 10.1016/j.jallcom.2016.03.240

[B28] Shi L. Li Y. Cai X. Zhao H. Lan M. (2017). ZIF-67 derived cobalt-based nanomaterials for electrocatalysis and nonenzymatic detection of glucose: difference between the calcination atmosphere of nitrogen and air. J. Electroanal. Chem. 799, 512–518. 10.1016/j.jelechem.2017.06.053

[B29] Shi L. Zhu X. Liu T. Zhao H. Lan M. (2016). Encapsulating Cu nanoparticles into metal-organic frameworks for nonenzymatic glucose sensing. Sensors Actuators B Chem. 227, 583–590. 10.1016/j.snb.2015.12.092

[B30] Song Z. Cheng N. Lushington A. Sun X. (2016). Recent progress on mof-derived nanomaterials as advanced electrocatalysts in fuel cells. Catalysts 6:116 10.3390/catal6080116

[B31] Sonkar P. K. Ganesan V. (2015). Synthesis and characterization of silver nanoparticle-anchored amine-functionalized mesoporous silica for electrocatalytic determination of nitrite. J. Solid State Electrochem. 19, 2107–2115. 10.1007/s10008-014-2725-3

[B32] Taylor C. Kenausis G. Katakis I. Heller A. (1995). “Wiring” of glucose oxidase within a hydrogel made with polyvinyl imidazole complexed with [(Os-4,4′-dimethoxy-2,2′-bipyridine)Cl]+/2+1. J. Electroanal. Chem. 396, 511–515. 10.1016/0022-0728(95)04080-8

[B33] Wang J. Han G. Wang L. Du L. Chen G. Gao Y.. (2018). ZIF-8 with ferrocene encapsulated: a promising precursor to single-atom fe embedded nitrogen-doped carbon as highly efficient catalyst for oxygen electroreduction. Small 14:1704282. 10.1002/smll.201704282 29504246

[B34] Xie Y. Song Y. Zhang Y. Xu L. Miao L. Peng C. (2018). Cu metal-organic framework-derived Cu Nanospheres@Porous carbon/macroporous carbon for electrochemical sensing glucose. J. Alloys Compd. 757, 105–111. 10.1016/j.jallcom.2018.05.064

[B35] Xu F. Cui K. Sun Y. Guo C. Liu Z. Zhang Y.. (2010). Facile synthesis of urchin-like gold submicrostructures for nonenzymatic glucose sensing. Talanta 82, 1845–1852. 10.1016/j.talanta.2010.07.087 20875586

[B36] Yadav D. K. Ganesan V. Marken F. Gupta R. Sonkar P. K. (2016). Metal@MOF materials in electroanalysis: silver-enhanced oxidation reactivity towards nitrophenols adsorbed into a zinc metal organic framework—Ag@MOF-5(Zn). Electrochim. Acta 219, 482–491. 10.1016/j.electacta.2016.10.009

[B37] Yang L. Xu C. Ye W. Liu W. (2015). An electrochemical sensor for H_2_O_2_ based on a new Co-metal-organic framework modified electrode. Sensors Actuators B Chem. 215, 489–496. 10.1016/j.snb.2015.03.104

[B38] Yang N. Guo K. Zhang Y. Xu C. (2020). Engineering the valence state of ZIF-67 by Cu_2_O for efficient nonenzymatic glucose detection. J. Mater. Chem. B 8, 2856–2861. 10.1039/D0TB00094A 32186316

[B39] Yin D. Liu J. Bo X. Li M. Guo L. (2017). Porphyrinic metal-organic framework/macroporous carbon composites for electrocatalytic applications. Electrochim. Acta 247, 41–49. 10.1016/j.electacta.2017.06.176

[B40] Zhang E. Xie Y. Ci S. Jia J. Wen Z. (2016). Porous Co3O4 hollow nanododecahedra for nonenzymatic glucose biosensor and biofuel cell. Biosens. Bioelectron. 81, 46–53. 10.1016/j.bios.2016.02.027 26918617

[B41] Zhang X. Xu Y. Ye B. (2018). An efficient electrochemical glucose sensor based on porous nickel-based metal organic framework/carbon nanotubes composite (Ni-MOF/CNTs). J. Alloys Compd. 767, 651–656. 10.1016/j.jallcom.2018.07.175

[B42] Zhou H. Zheng M. Tang H. Xu B. Tang Y. Pang H. (2020). Amorphous intermediate derivative from zif-67 and its outstanding electrocatalytic activity. Small 16:1904252. 10.1002/smll.201904252 31821688

[B43] Zhou K. Mousavi B. Luo Z. Phatanasri S. Chaemchuen S. Verpoort F. (2017). Characterization and properties of Zn/Co zeolitic imidazolate frameworks vs. ZIF-8 and ZIF-67. J. Mater. Chem. A 5, 952–957. 10.1039/C6TA07860E

[B44] Zhu N.-N. Liu X.-H. Li T. Ma J.-G. Cheng P. Yang G.-M. (2017). Composite system of ag nanoparticles and metal–organic frameworks for the capture and conversion of carbon dioxide under mild conditions. Inorg. Chem. 56, 3414–3420. 10.1021/acs.inorgchem.6b02855 28263612

[B45] Zhu Q. L. Xu Q. (2014). Metal-organic framework composites. Chem. Soc. Rev. 43, 5468–5512. 10.1039/C3CS60472A 24638055

[B46] Zhu R. Ding J. Yang J. Pang H. Xu Q. Xu Q.. (2020). Quasi-ZIF-67 for Boosted oxygen evolution reaction catalytic activity via a low temperature calcination. ACS Appl. Mater. Interfaces 12, 25037–25041. 10.1021/acsami.0c05450 32378882

